# Impact of Different Types of Physical Exercises for the Management of Older Women with Urinary Incontinence: A Systematic Review of Randomized Clinical Trials

**DOI:** 10.3390/jcm14103425

**Published:** 2025-05-14

**Authors:** Waleska Souza da Rocha, Aline Reis-Silva, Ana Carolina Coelho-Oliveira, Marcia Cristina Moura-Fernandes, Rosane da Silva Alves Cunha, Ayman Alhammad, Danúbia da Cunha de Sá-Caputo, Mario Bernardo-Filho, Redha Taiar

**Affiliations:** 1 Medicina Laboratorial e Tecnologia Forense, Universidade do Estado do Rio de Janeiro, Rio de Janeiro 20551-030, RJ, Brazil; waleska.access@gmail.com; 2 Laboratório de Vibrações Mecânicas e Práticas Integrativas, Departamento de Biofísica e Biometria, Instituto de Biologia Roberto Alcântara Gomes and Policlínica Universitária Piquet Carneiro, Universidade do Estado do Rio de Janeiro, Rio de Janeiro 20551-030, RJ, Brazil; fisio.alinereis@hotmail.com (A.R.-S.); anacarol_coelho@hotmail.com (A.C.C.-O.); marciafernandesfisio@hotmail.com (M.C.M.-F.); rosane66rj@hotmail.com (R.d.S.A.C.); drdanubia@gmail.com (D.d.C.d.S.-C.); bernardofilhom@gmail.com (M.B.-F.); 3 Programa de Pós-Graduação em Ciências Médicas, Faculdade de Ciências Médicas, Universidade do Estado do Rio de Janeiro, Rio de Janeiro 20551-030, RJ, Brazil; 4 Department of Physiotherapy, College of Medical Rehabilitation Sciences, Taibah University, Al-Madinah Al-Munawarrah 41477, Saudi Arabia; aalhamad@taibahu.edu.sa; 5 Université de Reims, Department of Sport Sciences, MATériaux et Ingénierie Mécanique (MATIM), 51687 Reims, France

**Keywords:** urinary incontinence, pelvic floor muscle, quality of life, physical performance

## Abstract

**Background/Objectives:** Urinary incontinence (UI) is particularly prevalent in women of all ages, but especially in older women, due to risk factors that contribute to pelvic floor muscle weakness. Urinary incontinence can have a significant psychosocial impact and compromise the quality of life of affected women. It is reported that physical activity seems to be inversely associated with UI. The aim is to summarize the impact of different modalities of physical exercises in management of older women with UI. **Methods**: Searches were performed in the databases PubMed, Web of Science, EMBASE and Scopus. The searches were performed on 10 December 2024. Only randomized clinical trials were included. Ninety-three papers were initially screened, and five full papers reached all the inclusion criteria describing the effects of exercise on urinary incontinence. The studies included a total of 352 elderly participants aged 60 years or older. Of these participants, 268 lived in nursing homes and 84 lived in a local community. The intervention period varied among the selected studies from 6 weeks to 12 months, and in general, the pelvic floor training was used. **Results**: Improvements in the quality of life and in the physical performance of the older women with UI were reported, although the risk of bias was classified as “some concerns”. **Conclusions**: Moreover, it is revealed that different types of exercises are used to “work” the pelvic floor muscle and contribute, in general, to reducing the symptoms, and improving the quality of life of older women with UI due to the positive impact of the exercises.

## 1. Introduction

During the aging process, organs generally begin to progressively lose some functions [1]. These changes due to aging occur in the body’s cells, tissues, and organs, and these can disrupt the functioning of bodily systems in both men and women [2]. There are some disorders due to aging that affect both sexes, with particularities related to each sex and different impacts, such as urinary incontinence (UI) [3,4].

UI is defined as the involuntary loss of urine, often triggered by sudden increases in intra-abdominal pressure, such as during coughing or sneezing, or associated with urinary urgency [5]. The main types of UI are: stress UI caused mainly by PFM weakness; urge UI caused by detrusor overactivity; and mixed UI, which occurs due to a combination of the symptoms of stress and urge incontinence [5]. UI is particularly prevalent in women of all ages, but especially in postmenopausal women, due to risk factors such as advanced age, previous pregnancies, and accumulation of body fat leading to obesity [6]. These factors contribute to pelvic floor muscle weakness, which, combined with other mechanisms such as hormonal changes related to menopause, recurrent urinary tract infections, and metabolic and neurological diseases, contribute to the development of UI [7].

UI imposes a psychosocial burden and is strongly associated with decreased health-related quality of life among affected women [8]. Women with UI often experience social embarrassment, anxiety, and depression, further compromising their overall well-being [9]. In consequence, self-esteem is affected, and the functional independence and activities of daily living are committed [8]. Treatments for UI can differ; while for urge UI the first-line treatment is medication, treatment for stress UI advocates exercise [6].

Regular physical activity is generally recommended to prevent disease and promote health during aging [10]. Various types of exercise can be performed at different stages of life to prevent and treat disease [11]. In this context, Townsend et al., 2008 reported that a regular practice of moderate and long-term physical activity is inversely associated with the prevalence of urinary incontinence [12]. Evidence suggests that increased levels of physical activity can contribute to overall body strengthening, promote recruitment of the pelvic floor musculature, and favor the reduction in body weight, factors that, together, can alleviate the symptoms of urinary incontinence [13]. Lee and Hirayama et al., 2012 reported that habitual walking decreased the odds of UI by approximately half in older women of various ethnic backgrounds [13].

Other types of exercises, such as whole-body vibration exercises (WBVE), seem to improve the pelvic floor muscle strength and quality of life in patients with UI (Farzinmehr et al., 2015) [14]. Furthermore, the literature has reported that yoga and pilates also improve clinical conditions and balance exercises, such as Tai Chi, have also been suggested for the treatment of women with UI [15,16,17].

Pelvic floor muscle training (PFMT) is widely accepted and has shown promising results for the treatment of UI [18]. Pelvic PFMT is defined as a therapeutic intervention based on physical exercises, with the purpose of promoting improvements in power, strength, resistance, and relaxation of the functional components of the pelvic floor muscles [19]. PFMT might involve specific and varied exercises to strengthen the muscles that support the bladder, uterus, and bowel, thereby increasing control over urination [20]. PFMT requires voluntary contraction of the pelvic floor muscles, that spanning the outlet of the pelvis from side to side and front to back [21]. When contracted correctly, there is cranio-ventral movement that contributes to lifts and squeezes around the vagina, urethra, and anus [22]. In many PFMT, it is asked of the women to squeeze and lift the pelvic floor muscle. Other types of exercise (abdominal, hip, or respiratory) may also be included in the PFMT [23]. In addition, Gonzaga et al., 2024 reported that women may include voluntary pelvic floor muscle contractions in an existing exercise routine such as Pilates [15].

Exercise not only helps to reduce the frequency of UI, but also contributes to an overall improvement in the quality of life of affected women of all ages [24] and in the functionality [25]. Furthermore, exercises are cost-effective, non-invasive, and can be performed without specialized equipment, making them accessible to a wide range of women, especially older adults [26]. These considerations associated with the negative consequences that the UI brings to the life of an individual have stimulated the realization of the current systematic review. The aim is to evaluate the impact of different modalities of physical exercises on the management of older women with urinary incontinence.

## 2. Material and Methods

The proposal of this systematic review was registered in the International Prospective Register of Systematic Reviews (PROSPERO) under the registration number CRD42024543002 [27]. This systematic review followed the Preferred Reporting Items for Systematic Review and Meta-Analysis Protocols (PRISMA) Statement [28].

The current systematic review aimed to answer the question: Which is the impact of different modalities of physical exercises on the management of older women with urinary incontinence? The PICOS strategy was used to determine the components of the research question: P—Participants = older women with urinary incontinence; I—Interventions = different modalities of exercises; C—Comparison = modality of exercises, or with a control group; O—Outcome = impact on the urinary incontinence; S—Study design = randomized clinical trials [29].

### 2.1. Eligibility Criteria

Inclusion criteria consisted of original randomized studies on urinary incontinence in elderly women with interventional studies with inter- or intra-group comparisons, independent of the language, and year of publication.

Exclusion criteria were: (i) review articles; (ii) conference abstracts; (iii) animal studies; (iv) case reports; (v) therapeutic interventions using electrical devices; (vi) studies with individuals under 60 years of age; (vii) studies involving pharmacological approaches; or (viii) surgical interventions.

### 2.2. Search Strategy

Two authors independently conducted the search strategy (ARS and WSR). An extensive literature search was performed using recognized electronic biomedical science databases and by hand-searching reference lists of articles that specifically investigated the effects of exercise on urinary incontinence. This search was applied to the following electronic databases: PubMed, Web of Science, EMBASE and Scopus. The searches were performed on 10 December 2024.

The strings used in the searches in each database were: (“older women” OR “older woman” OR “elderly female*”) AND (“urinary Incontinence” OR “urge incontinence” OR “stress incontinence” OR “involuntary urine”) AND (exercise* OR “physical activity*” OR “physical exercise*”) AND “randomized clinical trial”.

### 2.3. Study Selection and Data Extraction

After exporting all publications found in the databases to the Rayyan—Intelligent Systematic Reviews website (https://www.rayyan.ai/ accessed on 1 January 2025), duplicate records were removed [30]. Two authors (ARS and WSR) independently read the titles and abstracts according to the inclusion criteria and selected the studies for full reading. The researchers were blinded to each other’s decisions, and a third author (DCC) was available to resolve possible disagreements.

The data extracted from the included articles were imported into a Word table containing: (i) study information (author, year of publication); (ii) population/age (year) sample; (iii) results; (iv) intervention; (v) observed effects.

### 2.4. Risk of Bias

The Cochrane Risk of Bias 2 (RoB 2.0) assessment tool was used to assess methodological quality [31]. The RoB 2.0 tool involves a detailed assessment of risk of bias across multiple domains [32]. Each domain is assessed for its overall assessment, with specific questions scored as “yes”, “probably yes”, “probably not”, “no”, or “no information”. These individual domain lessons are then aggregated into an overall assessment of bias, categorized as “low”, “some concern”, or “high” based on a predefined algorithm [33]. To ensure the quality and robustness of the information, two researchers (ACC and MBF) independently assessed the RoB 2.0 tool [31]. They resolved any discrepancies through discussion with the third reviewer (DCC), reaching a final assessment by consensus.

## 3. Results

**Figure 1:**
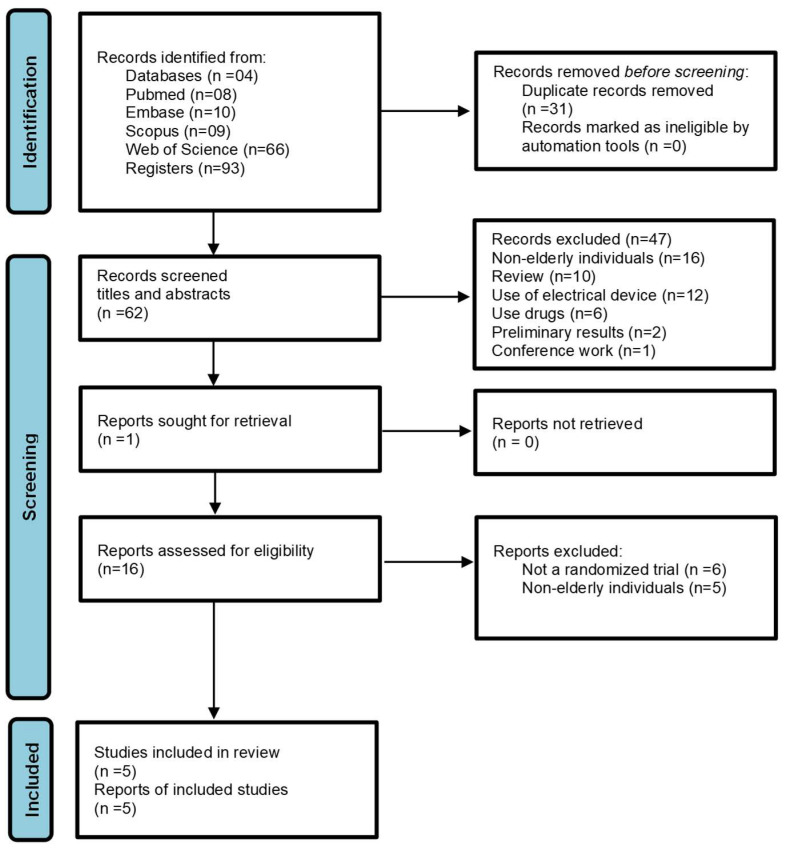
Search and screening strategy based on PRISMA 2020 flow diagram.

Ninety-three articles were initially found in the databases (MEDLINE/PubMed = 08, EMBASE = 10, Scopus = 09 and Web of Science = 66). After removing 31 duplicates, 62 records remained. Forty-seven publications were excluded after reading titles and abstracts. Sixteen full-text articles were read, and after careful analysis, 11 studies were excluded (six non-RCT and five non-elderly subjects). Five articles met all inclusion criteria describing the effects of exercise on women’s UI. The PRISMA flowchart shows all steps of the selection process (Figure 1) [28].

### 3.1. Study Characteristics

The five RCTs included in this review were published between 2008 and 2022 [34,35,36,37,38]. The studies included a total of 352 elderly participants aged 60 years or older. Of these participants, 268 lived in nursing homes and 84 lived in a local community.

All included studies assessed cognitive impairment, three by the Mini Mental Questionnaire [34,35,38], one Mini Cog questionnaire [37] and one the 14-item version of the Cognitive Screening Test [36]. Three studies [34,35,38] excluded participants with neurological diseases. Two studies excluded participants who were performing physiotherapy exercises for UI [35,37].

For the diagnosis of UI, Aslan et al., 2008, Sherburn et al., 2011, and Tak et al., 2012 [34,35,36] considered self-reporting with complaints of regular urinary enuresis, urgency, frequency or nocturia, while Chu et al., 2019 and Kannan et al., 2022 [37,38] used the ICIQ-SF questionnaire in addition to self-reporting.

The most used types of exercise in the included studies were PFMT [34,36,38]. Only one study used Pilates and yoga exercises [38], and two studies, in addition to pelvic floor training, also implemented general strength and balance training [36,37]. In addition, the bladder training (BT) was used [34,36,37].

The intervention period varied among the selected studies from 6 to 8 weeks [34,37,38], 22 weeks [36] and 12 months [35].

Additionally, Aslan et al., 2008 included women with regular complaints of urinary enuresis, urgency, frequency or nocturia [34]; Scherburn et al., 2011 included only women with stress urodynamic incontinence [35]; Tak et al., 2012 and Chu et al.2019 included women with complaints of mixed UI [36,37]; finally, Kannan et al., 2022 included women with stress or mixed UI [38].

Information about the population regarding age and the number of individuals (n) in each selected study, outcomes, type of intervention, and observed impacts is presented in Table 1.

### 3.2. Intervention Protocols

The studies included in this review used PFMT, Kegel exercises, yoga, Pilates, flexibility exercises, muscle strength exercises, and balance as therapeutic exercises for UI. In the study published by Kannan et al., 2022 [38], Virabhadrasana and Parsvakonasana poses (squeezing the heels toward the midline, which creates a sensation of lifting in PFMT were performed in the Yoga group, and in the Pilates group, Conventional treatment consisted of performing exercises initially in positions of least resistance to gravity (lateral, dorsal, ventral decubitus or quadruped position), with progression to positions of greater gravitational resistance (sitting and standing positions). The tasks involved contraction of the urethral muscles, voluntary control of evacuation (or gas emission) and the combination of contraction of the urethral meatus, evacuation control and elevation of the vaginal wall. The intervention protocols were heterogeneous, with different duration, intensity, training frequency, session duration, number of repetitions, rest time between repetitions and number of exercises.

### 3.3. Assessed Outcomes

The studies assessed several outcomes: Quality of Life [34,35,36]; ICIQ-UI [34,37,38]; UI severity [34,35,37,38]; PFMT evaluated by digital palpation [34]; Physical performance and Risk of fall [35,36,37]; Daily urinary forms [35,36]; Urinary symptoms [34,37]; Bother score (VAS) [35].

### 3.4. Quality of Life

Aslan et al., 2008 assessed quality of life using the King’s Health Questionnaire, in which higher scores indicate worse quality of life related to each domain [34]. The authors observed a significant reduction (*p* = 0.05) in the scores related to emotional aspects, general health perception and physical limitations after 8 weeks of intervention, compared to the values obtained in the pre-treatment period. When the “impact of incontinence” subdivision was considered in each of the three observations, the scores were statistically lower for the treatment group compared to the control group (*p* = 0.05); there was also a significant difference for the treatment group in the “sleep/energy” subdivision in the pre-treatment scores for 8 weeks and in the scores from 8 weeks to 6 months (*p* = 0.000) (intervention vs. control).

Sherburn et al., 2011 investigated quality of life using the Assessment of Quality of Life (AQoL) instrument and found that there was no significant difference between the two groups in the global measure of health-related quality of life at the end of the intervention [35]. Similarly, no differences were observed between the PFMT and BT groups in any of the specific domains assessed by the questionnaire. Tak et al., 2012 assessed quality of life using two questionnaires, the SF-12 questionnaire and the Incontinence Quality of Life Instrument (I-QOL) and did not observe any statistical difference for any of the groups analyzed (intervention vs. control) [36].

### 3.5. UI Severity

Chu et al., 2019 assessed UI severity scores using the ICIQ-UI and found significant improvements in relation to baseline in the experimental group (*p* = 0.04) [37]. Kanann et al., 2022 [38] applied the ICIQ-SF questionnaire and, in the intragroup analysis of the scores, identified a significant effect of the three interventions from baseline to the fourth week (*p* < 0.01), from the fourth to the twelfth week (*p* < 0.05) and from baseline to the twelfth week (*p* < 0.01). The between-group analysis revealed that yoga practice was significantly more effective than the Pilates method (*p* = 0.02) in improving urinary incontinence symptoms, considering the comparison between yoga, Pilates and PFMT. Aslan et al., 2008 [34] found that, in the pad tests performed by the intervention group, 24% of the participants presented urinary losses classified as moderate to intense (11–59 g), while in the control group this proportion was 16%. The mean urine loss was 7.12 ± 12.07 g in the treatment group and 8.20 ± 14.13 g in the control group. However, no statistically significant differences were observed between the groups in the results of the pad test (*p* = 0.13). Although at the end of the intervention the group submitted to PFMT presented lower volumes of urine loss during the cough stress test compared to the BT group, this difference also did not reach statistical significance.

In the study by Kannan et al., 2022 [38], the intragroup analysis showed a statistically significant reduction in urinary loss (in grams), assessed by the pad test, from baseline to the fourth week in the yoga and PFMT groups (*p* < 0.01), while in the Pilates group this reduction was not significant. However, the decrease in urinary loss from baseline to the twelfth week was significant in all three groups (*p* < 0.01). The intergroup analysis of the pad test data indicated that there was no significant effect of the time-group interaction between yoga, Pilates and PFMT, as well as between yoga and Pilates (*p* > 0.05).

### 3.6. Pelvic Floor Muscle Training (Evaluated by Digital Palpation)

Aslan et al., 2008 [34] demonstrated that PFM strength, assessed by digital palpation in the assessments performed after 8 weeks and at 6 months, showed a significant increase in the intervention group compared to the control group (*p* = 0.000). Furthermore, a significant improvement in muscle strength was observed between the 8-week and 6-month periods in the intervention group (*p* = 0.005).

### 3.7. Physical Performance and Risk of Fall

According to Sherburn et al., 2011 [35], the PFMT and BT groups did not show any significant difference in the TUG test.

### 3.8. Daily Urinary Forms

In the study by Sherburn et al., 2011 [35], patients undergoing PFMT reported fewer episodes of urinary incontinence compared to the BT group, as recorded in a 7-day accident diary at the end of the intervention. The data indicate a 52.9% reduction in the frequency of incontinence episodes in the PFMT group, in contrast to a 36.7% reduction observed in the BT group. Tak et al., 2012 [36]. demonstrated a reduction in voiding frequency, as recorded in a voiding diary, at 3 and 6 months of follow-up in both the fitness program group and the control group. However, no statistically significant differences were observed between the groups. Similarly, the incidence of urinary incontinence episodes showed a decrease at 3 months and a 51% reduction at 6 months in both groups, also with no significant differences between the interventions [36].

#### 3.8.1. Urinary Symptoms

Aslan et al., 2008 [34] identified statistically significant reductions in symptoms of urinary urgency and increased urinary frequency in the group undergoing therapeutic intervention, compared to the control group, between the baseline assessment and the 8-week and 6-month follow-up periods (*p* ≤ 0.05). However, no significant differences were observed between the outcomes assessed at weeks 8 and 24 (*p* > 0.05). Additionally, a significant reduction in nocturia episodes was observed in the treatment group, relative to the control, between the 8-week and 6-month assessment points (*p* ≤ 0.05).

#### 3.8.2. Bother Score (VAS)

Sherburn et al., 2011 [35] demonstrated that, at the end of the intervention, participants in the PFMT group reported a significantly lower degree of discomfort related to urinary symptoms, when compared to the group that received BT (*p* = 0.009). The average reduction in the perception of discomfort, measured using a 10 cm visual analog scale (VAS), was 2.5 cm in the PFMT group, while in the BT group the average reduction was 1.6 cm throughout the intervention period.

#### 3.8.3. Risk of Bias

**Figure 2:**
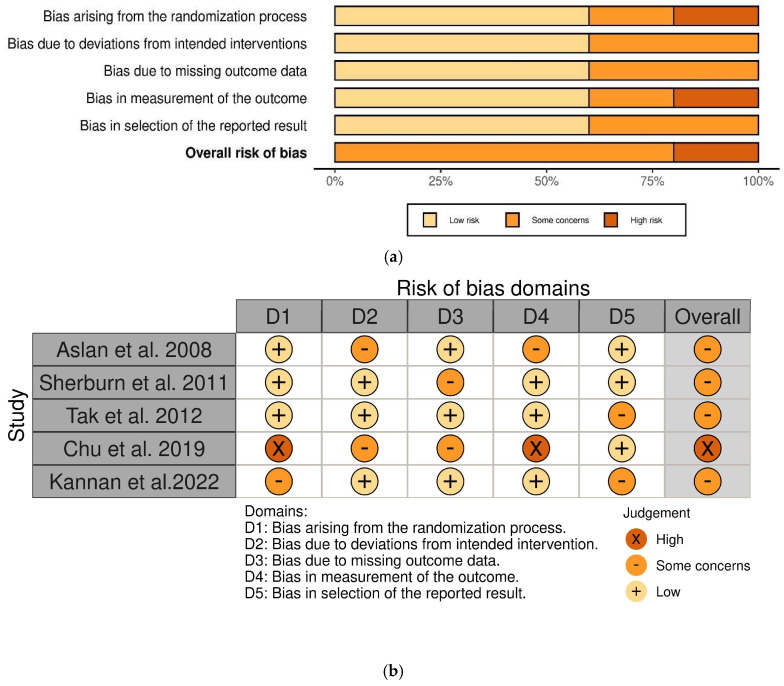
Risk of bias assessment of the studies included in this review using RoB 2. (**a**) The figure shows the evaluation of the quality of the included studies; dark brown = high; light brown = low; orange = some concerns; (**b**) The figure presents an assessment of the quality of the included studies and the risk of bias: “+” means low risk of bias; “×” means high risk of bias; “–” some concerns [34,35,36,37,38].

The risk of bias of the included studies is shown in Figure 2a,b according to the RoB 2 [31], and, in the current study, the risk of bias assessments was based on primary data from the selected articles.

### 3.9. Performance Bias

#### 3.9.1. Allocation

Three studies [34,35,36] used an acceptable method of generating the expected sequence (e.g., computer-generated random number sequences or envelopes), so we rated the risk of bias as low. One study [37] used block randomization; Kannan et al., 2022 did not specify details of how the randomization was conducted, and we considered the risk of selection bias to be a concern [38].

#### 3.9.2. Assessments of Results

Three studies [34,36,38] provided satisfactory information regarding the segmentation of assessors and results and were judged as low risk; two studies [35,37] did not show clear information, and objective results we classified as “some concerns”.

## 4. Discussion

Urinary incontinence is prevalent in older individuals, with a high prevalence among women. Non-surgical and non-pharmacological interventions are particularly desirable for the treatment of these women [39]. Among non-pharmacological interventions, physical exercise is of particular interest [40]. This systematic review summarizes the impact of different types of physical exercise on the management of elderly women with urinary incontinence.

PFMT is widely accepted as an effective intervention and is recommended in four publications [34,35,36,38]. Aslan et al., 2008 cited the use of Kegel exercises that could be considered PFMT [34,41]. Both Aslan et al., 2008 and Sherburn et al., 2011 demonstrated significant improvement in UI following a PFMT protocol [34,35]. However, Tak et al., 2012 showed no significant difference with a PFMT protocol compared to the control group [36]. Sigurdardottir et al., 2019, in agreement with Aslan et al., 2008 and Sherburn et al., 2011 [34,35], also demonstrated significant benefits in UI following PFMT in women [42]. Although all studies performed the PFMT, the divergence of results in relation to the study by Tak et al., 2008 may be due to the population evaluated in this study, which, in addition to being elderly, had at least one chronic disease associated with UI and were dependent on caregivers [36]. Additionally, urinary complaints in the study by Tak et al., 2008 were assessed only indirectly and subjectively by means of a questionnaire, which could lead to subjective or less accurate information than the pad test or the digital pelvic floor muscle strength test that were used in the other studies [36].

Chu et al., 2019 [37] used a validated video exercise program, FlexToBa (short for flexibility, toning, and balance). Chu et al., 2019 [37], reported a significant improvement in urinary symptoms in the group that performed the FlexToBa protocol; however, they only showed intragroup analysis and the only measure of UI assessment was the ICIQ SF questionnaire. Considering the findings presented by Chu et al., 2019, other possibilities of physical exercises can collaborate in the treatment of UI. However, it would be important to perform intergroup analyses with a control group and use specific assessment measures, such as pad tests, and not subjective ones [37].

Although there was an important heterogeneity in the exercises among the studies selected in the current review, PFMT was used in majority of the studies, as well as Dumoulin et al., 2018 [6], who reported a cure or reduction in undesirable symptoms of UI and all other types of UI due to PFMT, except in Chu et al., 2019 [37].

PFMT has demonstrated effectiveness in reducing the frequency of urinary leakage episodes, the volume of leakage assessed through standardized tests with pads in [42] a clinical environment, as well as in attenuating the symptoms measured by specific instruments for assessing urinary incontinence [6,24,43]. Furthermore, it is suggested that PFMT could be included in first-line conservative treatment programs for women with UI [44,45,46]. However, there is a relevant issue related to PFMT that is cited in this current systematic review, which is the wide variety of exercises in PFMT.

The studies that included PFMT as an intervention measure performed different protocols, including the positioning adopted during the exercises (lying, sitting, lateral decubitus, standing), the intensity of the contraction (intense, moderate), the time of contraction and rest, and in some studies did not clearly describe the number of times to be performed during the day or how many times per week. The variety of exercises in PFMT may justify the need for protocols with such different intervention periods, from 6 weeks to 12 months. Furthermore, the variety of responses to the intervention may be associated with the protocols used for PFMT.

In addition to using PFMT, the BT has been reported to help individuals gain bladder control and reduce UI, and it involves establishing a routine for urinating even when there is no urge. Todhunter-Brown et al., 2022 [47] agreed with four publications in this review where the BT has been used in conjunction with PFMT (Aslan et al., 2008, Sherburn et al., 2011, Tak et al., 2012 and Chu et al., 2018) [34,35,36,37]. Aslan et al., 2008 [34] reported significant decreases in urinary urgency, urinary frequency, and nocturia following a (PFMT + BT) protocol, while Sherburn et al., 2011 [35] compared a BT vs. PFMT protocol in their study and observed that the group that performed PFMT obtained more significant benefits in urinary symptoms than the group that performed BT. The study by Chu et al., 2018 [37] demonstrated a significant improvement in urinary symptoms but not in nocturia with the “FlexToBa” + BT protocol. Finally, Tak et al., 2012 did not show any significant benefit with the use of a protocol (PFMT + BT + Incondition program) in urinary symptoms [36]. Overall, these findings may suggest that it seems that patients respond better to BT when used in combination with PFMT. Furthermore, considering that urge UI is more prevalent in older women, the included studies do not clearly demonstrate an improvement in symptoms of urgency, urinary frequency and nocturia. Perhaps this profile of older individuals with greater muscle weakness responds better to exercise therapies than behavioral therapies for UI. According to Yıldız N and Özlü A 2022, a young age and high levels of education are predictive factors for successful treatment with BT in women with UI [48].

Regarding the quality of life, according Astrom et al., 2021, UI significantly reduces the quality of life of women [49]. UI can restrict social life, such as going out or traveling and even going to work [50]. In addition, women who have these complaints usually do not talk about it with anyone, not even their spouse, because they are embarrassed by the smell of urine or being wet in public [9].

Aslan et al., 2008 [34] assessed quality of life using the King’s Health Questionnaire and demonstrated a significant decrease in scores for general health perception, physical limitations, emotions, and “impact of incontinence” and “sleep/energy”. However, Sherburn et al., 2011 and Tak et al., 2012 [35,36] found no significant difference in the measure of health-related quality of life at the end of the intervention. This difference in results can probably be attributed to the relationship between the improvement in UI and the tool used to assess quality of life in these studies. Aslan et al., 2008 showed improvement using the King’s Health Questionnaire, a specific questionnaire to assess quality of life in UI [34]; Sherburn et al., 2011 [35], although demonstrating a substantial improvement in UI, used a generalist and non-specific tool to assess the quality of life in UI (Assessment of Quality of Life (AQoL). Tak et al., 2012, although using two questionnaires to assess quality of life (one specific to UI and one not), did not demonstrate a significant change in the quality of life [36]. This may have been because the participants did not show significant improvement in UI. Raddaha et al., 2022 [51], in agreement with Aslan et al., 2008 [34], also reported significant benefits in UI and quality of life in women and used a specific questionnaire to assess the quality of life in women with UI.

### 4.1. Limitations of the Study

Firstly, the findings of the current systematic review must be interpreted with caution. Although four databases were used in the searches, probably more RCTs could be found if other databases were searched. The same could be considered regarding the search terms; although inclusive, if a broader search strategy was used, more studies might have been identified. Also, another point is that of the studies included, only Sherburn et al., 2011 performed urodynamic evaluation to diagnose the types of urinary incontinence [35]. Furthermore, in view of the diversity of the selected publications, which differed in study focus (e.g., type of exercise, repetitions, intervention period), and in view of significant methodological heterogeneity among the studies, the readers must take care in generalizing the findings. In consequence, the risk of bias was classified as “some concerns”.

### 4.2. Strengths of the Study

Although the limitations of the studies should be considered, the present systematic review summarizes the impact of different types of physical exercises that can be used to improve PFM functioning in elderly women with UI and thus result in an improved quality of life for these women.

## 5. Conclusions

Based on the results of this systematic review, it can be concluded that physical exercise plays a significant role in improving PFM function. In elderly individuals, both bladder training (BT) and pelvic floor muscle training (PFMT) are conservative strategies used in the management of urinary incontinence. However, the available evidence suggests that PFMT has shown more consistent results in improving urinary symptoms. Consequently, these improvements lead to a better quality of life and have a significant positive impact in the clinical context.

## Figures and Tables

**Table 1:** Population/age, the number of individuals in each selected study, outcomes, type of intervention, and observed impacts.

Author	Population/Age (Years Old)	Groups/Sample (n)	Outcomes	Intervention	Observed impacts
Aslan et al., 2008 [34]	Living at a rest home/70–89	Treatment Group: 25 Control Group: 25	- QOL Scale Test. - The King’s Health Questionnaire. - 1 h pad test. - PFM muscle strength evaluated by digital palpation. - Rankin Scale evaluated the functional condition - Daily urinary forms	Intervention 6–8 weeks, BT, and Kegel exercises Control: BT and the PFMT were not performed	- In the group undergoing therapeutic intervention, a reduction in complaints of urinary urgency (52%), decreased urinary frequency (64%) and episodes of nocturia (32%) were observed at the end of treatment. - PFM was found to be strengthened by 56% after treatment - Quality of life (QOL) scores showed statistically significant improvement in all dimensions assessed by the scale after therapeutic intervention.
Sherburn et al., 2011 [35]	Community-dwelling women/over 65	PFMT group: 43 BT group: 41	- Urine loss on a stress test. - ICIQ-UI SF. - Global rating of change. - 7-day accident diary (recording on a form the number of leakage episodes during the seven days). - “Bother” VAS; - Assessment QOL; - TUG. - Satisfaction with treatment. - Adherence to program.	PFMT group: 5-month exercise/session lasting 1 h, educational component and exercise for music class incorporating PFMT. Intensive PFMT, combining motor control, strength, endurance, power and functional training. BT Group: As in the PFMT group, each weekly group session began with an educational component followed by a gentle exercise for music class. Cognitive methods addressed the physiological control of the bladder and parameters related to bladder emptying, including guidance on skin care, adequate use of absorbent pads, management of water intake and adoption of the ideal posture during urination.	- The group undergoing PFMT demonstrated superior improvement, compared to the group treated with BT, in the cough stress test. - The pelvic PFMT group showed significantly more improvement in the ICIQ-UI SF scores at the end of the intervention. - A significant percentage difference was observed between the two groups in relation to stress urinary incontinence—characterized by the loss of urine during coughing or sneezing episodes—with an incidence of 48.8% in the PFMT group and 77.5% in the BT group. - The PFMT group reported a higher perceived improvement in overall urinary symptoms compared to the BT group. - At the end of the intervention, the group undergoing PFMT had a lower number of episodes of urinary leakage, as recorded in the seven-day accident diary, compared to the group treated with BT. - QOL: No differences were observed between the groups in any of the domains assessed by this questionnaire. - Bother VAS: At the end of the intervention, the group undergoing PFMT reported a lower degree of discomfort related to urinary symptoms compared to the BT group
Tak et al., 2012 [36]	Institutionalized older women	Intervention group: 85 Control group: 70	- Physical performance was measured with the PPT - Involuntary urine loss was assessed by recording a three-day voiding diary. Quality of life was assessed using two self-reporting instruments: the SF-12 questionnaire, which measures physical and mental health status in adults, and the I-QOL, which specifically assesses the impact of urinary incontinence on patients’ quality of life.	Intervention group: The Incondition program was structured in weekly 1 h training sessions for groups of 6 to 10 women over a 22-week period. Each session included behavioral and exercise training components. The behavioral intervention aimed to optimize urination control by improving knowledge about urinary continence, correcting behaviors related to using the bathroom (such as posture and relaxation techniques), and implementing BT and PFMT, with an emphasis on relaxation and breathing techniques. The exercise session, lasting 30 min, included warm-up activities, exercises aimed at improving upper limb mobility and hand function, as well as exercises involving getting up and sitting in a chair, walking and relaxation techniques. Control group: Participants received usual care, which included the use of pads to manage urinary incontinence and assistance with bathroom visits.	- All patients who started the Incondition program or usual care showed similar results in the primary and secondary outcome measures, except in relation to physical performance, in which a difference was observed between the groups. - The number of patients with UI showed a slight reduction in both the intervention and control groups. However, while in the control group the frequency of incontinence episodes showed a continuous decline over time, in the intervention group there was a slight increase after an initial reduction at three months. - Physical performance (PPT) improved in the intervention group. - The frequency of incontinence episodes in the intervention group reduced by 27% at three months and by 51% at six months, while in the control group the reductions were 24% and 42%, respectively. - PPT was superior in the intervention group, with an improvement of 13%, compared with a worsening of 4% in the control group. - No significant differences in quality of life (QOL) were observed between the groups.
Chu et al., 2019 [37]	Local community centers and geriatric clinics women/65 and older	Experimental group: 17 Control group: 16	- ICIQ-UI - Nocturnal Enuresis, and Sleep-interruption Questionnaire (NNES-Q); - FES-I assesses perception of balance and fear of falling. - The ABC scale assesses the fall risk.	Experimental group: Participants exercised three times a week using the “FlexToBa” (acronym for Flexibility, Tone, and Balance) home exercise program, consisting of six exercise modules distributed on digital media (DVDs) for progression over 24 weeks. In addition, a DVD for suppressing urinary urgency was used, associated with behavioral measures, both developed by a specialized nurse. A home visit was conducted in which a trained research coordinator provided written recommendations for adaptations to the home environment, based on a checklist for fall prevention. Control group: Participants in the usual care group were instructed to schedule an appointment with a urinary incontinence specialist or with a physical therapist/nurse specialized in UI rehabilitation.	- UI severity scores showed significant improvement in relation to baseline values in the experimental group, with this improvement being greater than that observed in the control group. No significant differences were identified between the groups regarding the improvement of nocturia or nocturnal enuresis. - At the end of the test, the fall risk score showed no significant difference between the groups. - No differences were observed between groups regarding changes in physical activity levels or sedentary behavior at follow-up.
Kannan et al., 2022 [38]	Elderly care centres/60 or above,	Yoga: 10 Pilates: 10 Standard care control: 10	- ICIQ-SF; - 1 h pad test with a stress test.	Supervised sessions once a week for four weeks, followed by unsupervised home exercises guided by a CD for eight weeks. Yoga group: Virabhadrasana and Parsvakonasana poses plus hatha style yoga, which included eight poses: Tadasana, Utkatasana, Trikonasana, Malasana, Viparita Karani Variation, Salamba Setu Bandhasana, Supta Baddha Konasana, and Savasana. Pilates Group: exercises targeting the pelvic floor and core muscles (transverse abdominis and multifidus). Exercises included Pilates breathing, knee swings, heel slides, pelvic clock, tailbone flexion, pelvic lift, roll down, leg springs, circles, single leg stretch, scissors, spinal stretch, and swan prep. Eight contractions were performed for each of these PFMT (24 contractions in each session). Each contraction lasted 5–6 s, followed by relaxation for 10 s. PFMT group: group initially performed exercises in antigravity positions (lateral, dorsal, ventral or quadrupedal decubitus) with subsequent progression to counter gravity positions (sitting and standing). Activities included urethral contraction, evacuation control and a combination of urethral orifice contraction, evacuation control and vaginal wall elevation. Each session involved eight sets of PFM exercises, totaling 24 contractions per session. Each contraction was held for five to six seconds, followed by a 10 s relaxation period.	- Mean ICIQ-SF scores showed a progressive reduction in all three groups from baseline to weeks 4 and 12. Within-group analysis of ICIQ-SF scores revealed a significant effect of all three interventions in the period from baseline to weeks 4 and 12, as well as between weeks 4 and 12. - Between-group analysis indicated that yoga practice was more effective than the Pilates method in improving urinary continence, as assessed by the ICIQ-SF. - Intragroup analysis revealed a statistically significant reduction in urinary loss, assessed by the pad test, from the beginning of the study to the fourth week in the Yoga and PFMT groups (*p* < 0.01), while in the Pilates group no significant difference was observed (*p* > 0.05). However, the reduction in urinary loss from baseline to week 12 was statistically significant in all three groups (*p* < 0.01).

ABC scale: Activities Specific Balance Confidence Scale; BT: bladder training; Bother VAS: “Bother” visual analogue scale; DVD: Digital Versatile Disc; Falls Efficacy Scale International: FES I; ICIQ-UI SF: International Consultation on Incontinence Questionnaire; ICIQ-S: Short Form; I-QOL: Incontinence Quality of Life Instrument, NNES-Q: Nocturnal Enuresis and Sleep-interruption Questionnaire; PFM—pelvic floor muscle, PFMT: pelvic floor muscle training; PPT: physical performance test; QOL: Quality of life; SF12: 12-item Short Form Survey; VAS: visual analogue scale; UI: urinary incontinence; %: percentage; s: second; TUG: Timed Up and Go; “Flex Toba”: short for Flexibility, Toning, and Balance.

## Data Availability

The original contributions presented in the study are included in the article, further inquiries can be directed to the corresponding authors.
